# Glitter in the Darkness? Nonfibrillar β-Amyloid Plaque Components Significantly Impact the β-Amyloid PET Signal in Mouse Models of Alzheimer Disease

**DOI:** 10.2967/jnumed.120.261858

**Published:** 2022-01

**Authors:** Gloria Biechele, Laura Sebastian Monasor, Karin Wind, Tanja Blume, Samira Parhizkar, Thomas Arzberger, Christian Sacher, Leonie Beyer, Florian Eckenweber, Franz-Josef Gildehaus, Barbara von Ungern-Sternberg, Michael Willem, Peter Bartenstein, Paul Cumming, Axel Rominger, Jochen Herms, Stefan F. Lichtenthaler, Christian Haass, Sabina Tahirovic, Matthias Brendel

**Affiliations:** 1 Department of Nuclear Medicine, University Hospital of Munich, LMU Munich, Munich, Germany;; 2 German Center for Neurodegenerative Diseases Munich, Munich, Germany;; 3 Graduate School of Systemic Neuroscience, Ludwig-Maximilians-University Munich, Munich, Germany;; 4 Department of Neurology, Washington University, St. Louis, Missouri;; 5 Chair of Metabolic Biochemistry, Biomedical Center, Faculty of Medicine, LMU Munich, Munich, Germany;; 6 Munich Cluster for Systems Neurology, Munich, Germany;; 7 Department of Nuclear Medicine, Inselspital, University Hospital Bern, Bern, Switzerland;; 8 School of Psychology and Counselling and IHBI, Queensland University of Technology, Brisbane, Australia;; 9 Center of Neuropathology and Prion Research, University of Munich, Munich, Germany; and; 10 Neuroproteomics, School of Medicine, Klinikum Rechts der Isar, Technical University of Munich, Munich, Germany

**Keywords:** amyloid, fibrillar, nonfibrillar, PET signal, mouse

## Abstract

β-amyloid (Aβ) PET is an important tool for quantification of amyloidosis in the brain of suspected Alzheimer disease (AD) patients and transgenic AD mouse models. Despite the excellent correlation of Aβ PET with gold standard immunohistochemical assessments, the relative contributions of fibrillar and nonfibrillar Aβ components to the in vivo Aβ PET signal remain unclear. Thus, we obtained 2 murine cerebral amyloidosis models that present with distinct Aβ plaque compositions and performed regression analysis between immunohistochemistry and Aβ PET to determine the biochemical contributions to Aβ PET signal in vivo. **Methods:** We investigated groups of *App^NL-G-F^* and APPPS1 mice at 3, 6, and 12 mo of age by longitudinal ^18^F-florbetaben Aβ PET and with immunohistochemical analysis of the fibrillar and total Aβ burdens. We then applied group-level intermodality regression models using age- and genotype-matched sets of fibrillar and nonfibrillar Aβ data (predictors) and Aβ PET results (outcome) for both Aβ mouse models. An independent group of double-hit APPPS1 mice with dysfunctional microglia due to knockout of triggering receptor expression on myeloid cells 2 (Trem2^−/−^) served for validation and evaluation of translational impact. **Results:** Neither fibrillar nor nonfibrillar Aβ content alone sufficed to explain the Aβ PET findings in either AD model. However, a regression model compiling fibrillar and nonfibrillar Aβ together with the estimate of individual heterogeneity and age at scanning could explain a 93% of variance of the Aβ PET signal (*P* < 0.001). Fibrillar Aβ burden had a 16-fold higher contribution to the Aβ PET signal than nonfibrillar Aβ. However, given the relatively greater abundance of nonfibrillar Aβ, we estimate that nonfibrillar Aβ produced 79% ± 25% of the net in vivo Aβ PET signal in *App^NL-G-F^* mice and 25% ± 12% in APPPS1 mice. Corresponding results in separate groups of APPPS1/Trem2^−/−^ and APPPS1/Trem2^+/+^ mice validated the calculated regression factors and revealed that the altered fibrillarity due to Trem2 knockout impacts the Aβ PET signal. **Conclusion:** Taken together, the in vivo Aβ PET signal derives from the composite of fibrillar and nonfibrillar Aβ plaque components. Although fibrillar Aβ has inherently higher PET tracer binding, the greater abundance of nonfibrillar Aβ plaque in AD-model mice contributes importantly to the PET signal.

## Visual Abstract

**Figure:**
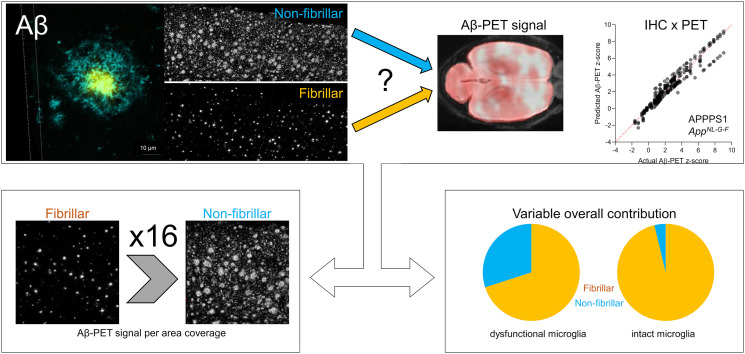

PET for β-amyloid (Aβ) is now widely used for identification and quantification of amyloidosis in the brain of suspected Alzheimer disease (AD) patients (*1*) and has been incorporated into the current research framework for diagnostic recommendations in AD (*2*). Here, the Aβ status (A) identified by PET serves for diagnosis, together with biomarkers for tau and neuronal injury (*2*). Furthermore, Aβ PET is used as an inclusion criterion in antiamyloid immunotherapy clinical trials (*3*) and as a progression biomarker for therapy evaluation in these trials (*4*). In the preclinical setting, Aβ PET has also become a useful tool for the dynamic assessment of neuropathology in transgenic Aβ mouse models (*5*,*6*). Despite the excellent correlation of Aβ PET with immunohistochemical gold standard assessments of amyloidosis in patients (*7*,*8*) and mouse models of AD (*6*,*9*), there has remained an uncertainty about the relative contributions of fibrillar and nonfibrillar Aβ components in plaques to the Aβ PET signal in vivo. This research gap needs to be closed because the 2 forms have differing neurotoxicity, and there is evidence that alterations in AD-related genes such as *TREM2* (triggering receptor expressed on myeloid cells 2) and *APOE *(apolipoprotein E) alter the net Aβ plaque fibrillarity, which would consecutively bias the relationship between plaque density and Aβ PET binding in vivo (*5*).

A human autopsy–validated ^18^F-florbetaben PET study showed preliminary evidence that diffuse plaques may make only a minor contribution to the net Aβ PET signal (*10*). However, autopsy-controlled data with ^18^F-flutemetamol in vivo (*11*) and comprehensive in vitro data (*12*) indicated that the binding of that structurally distinct tracer to diffuse plaques also contributes to the net PET signal. Furthermore, our recent preclinical study revealed a discernible Aβ PET signal in *App^NL-G-F^* mice (*13*), although this model displays only very limited fibrillar Aβ pathology (*14*). Therefore, we aimed to quantify the contributions of fibrillar and nonfibrillar plaque components to the Aβ PET signal in vivo in AD-model mice.

We recently demonstrated that the *App^NL-G-F^* and APPPS1 mouse models exhibit differences in Aβ plaque fibrillarity (*14*), such that a comparative study of these mice could help to determine the effect of fibrillarity on Aβ PET signal in vivo. Thus, we combined a standardized preclinical ^18^F-florbetaben PET study with immunohistochemical characterization of fibrillar versus nonfibrillar Aβ in *App^NL-G-F^* and APPPS1 mice examined at different pathologic stages. We then developed a regression model for immunohistochemistry and Aβ PET to establish the relative proportions of fibrillar and nonfibrillar sources in the Aβ PET signal in vivo. Furthermore, we validated the calculated regression factors in an independent cohort of APPPS1/Trem2^−/−^ and APPPS1/Trem2^+/+^ mice and tested a hypothesis that the nonfibrillar Aβ pool contributes more to the Aβ PET signal in APPPS1/Trem2^−/−^ mice than in APPPS1/Trem2^+/+^ mice.

## MATERIALS AND METHODS

### Experimental Design

All experiments were performed in compliance with the National Guidelines for Animal Protection, Germany, and with the approval of the regional animal committee (Regierung Oberbayern) and were overseen by a veterinarian. Animals were housed in a temperature- and humidity-controlled environment with a 12-h light–dark cycle and with free access to food (Sniff) and water. We conducted longitudinal ^18^F-florbetaben Aβ PET imaging in cohorts of female *App^NL-G-F^* (*n* = 18) and APPPS1 (*n* = 14) mice at 3, 6, and 12 mo of age, together with an age- and sex-matched group of wild-type (*n* = 8) mice. Of the Aβ mouse models, 56% had their baseline examination at 3 mo of age and the remaining 44% were imaged from 6 to 12 mo of age. All mice of each model originated from the same breeding colony. To exclude batch effects within each modality, we used separate cohorts of mice (*14*) for immunohistochemistry analyses of fibrillar and nonfibrillar Aβ plaque components in wild-type and AD-model mice (*n* = 3–4) at 3, 6, and 12 mo of age. We then applied intermodality regression models to separate the relative contributions of fibrillar and nonfibrillar Aβ plaque components to Aβ PET signals in the 2 strains.

### Animal Models

APP/PS1 (APPPS1-21) mice show extensive fibrillar Aβ plaque pathology, first evident at 6–8 wk of age (*15*). In contrast, *App^NL-G-F^ (App^NL-G-F/NL-G-F^*) is a murine model with relatively limited fibrillar Aβ plaque pathology but showing Aβ PET signal from 8 wk of age in homozygous mice (*16*,*17*). Wild-type controls were C57BL/6 mice.

### PET Imaging

#### PET Data Acquisition, Reconstruction, and Postprocessing

For all PET procedures, radiochemistry, data acquisition, and image preprocessing were conducted according to an established, standardized protocol (*6*). In brief, we obtained ^18^F-florbetaben Aβ PET recordings (average dose, 12.1 ± 1.8 MBq) with an emission window of 30–60 min after injection.

#### PET Image Analysis

We performed all analyses using PMOD (version 3.5; PMOD Technologies). Normalization of attenuation-corrected emission images to SUV ratio images was performed using previously validated periaqueductal gray matter (*18*) and white matter reference regions for the *App^NL-G-F^* and APPPS1 mouse models, respectively (*5*). We analyzed the wild-type mice separately with both reference regions to serve as controls for the Aβ mouse models. Bilateral neocortical volumes of interest (15 mm^3^) matching the region of interest in the immunohistochemistry analysis were applied for calculation of forebrain–to–white matter SUV ratio or forebrain–to–periaqueductal gray matter SUV ratio.

### Immunohistochemical Analysis

Groups of APPPS1 and *App^NL-G-F^* mice at an age of 3 mo (*n* = 4), 6 mo (*n* = 3), and 12 mo (*n* = 4) were transcardially perfused with ice-cold phosphate-buffered saline (0.1 M) followed by 4% paraformaldehyde, after being cryopreserved in 30% sucrose. The mouse tissue used for immunohistochemical analysis included some of the APPPS1 and *App^NL-G-F^* mouse brains used in our previous publication (*14*). All stainings and analyses were performed newly for the purpose of the present study. We collected 30-μm-thick coronal sections for free-floating immunostaining. We used the 3552 antibody (1:5,000 (*19*)) to label total Aβ, and we used thiazine red (2 μM; Sigma) to stain the fibrillar Aβ. Twenty-four images were acquired in 4 coronal sections (6 images per section) in regions matching PET using a confocal microscope (×20 dry objective, TCS SP5; Leica). Given the prominent differences in the levels of fibrillar Aβ between the APPPS1 and *App^NL-G-F^* mice, the confocal settings were optimized for each mouse model to acquire the thiazine red signal. For the 3- and 6-mo-old *App^NL-G-F^* mice, the averaging and accumulation confocal functions were set to 2, to better detect the thiazine red signal. An in-house–programmed macro from ImageJ (NIH) was used to analyze the total and fibrillar Aβ coverage.

As a validation analysis, we reanalyzed data from a previous study that included immunohistochemistry markers for fibrillar (x-34) and total (3552) Aβ components of Aβ plaques (*5*). Immunohistochemistry was obtained from APPPS1/Trem2^+/+^ and APPPS1/Trem2^−/−^ mice (3 and 6 mo, *n* = 4; 12 mo, *n* = 8). Aβ PET data were analyzed by the processing pipeline described above and at the same time points for both genotypes (APPPS1/Trem2^+/+^: three 3-mo-old, ten 6-mo-old, and ten 12-mo-old mice; APPPS1/Trem2^−/−^: seven 3-mo-old, nine 6-mo-old, and seven 12-mo-old mice). Furthermore, for validation purpose we obtained Aβ coverage for fibrillar (methoxy-x04 or x-34) and total (3552) Aβ components at 13 mo of age in the PET cohorts. In all datasets, nonfibrillar Aβ was calculated by subtraction of fibrillar Aβ from total Aβ (percentage nonfibrillar area = percentage total area − percentage fibrillar area).

### Statistics

Prism (version 8.43; GraphPad Software, LCC) was used for all statistical tests. A *P* value of less than 0.05 was considered to be significant for rejection of the null hypothesis.

#### Group-Level Analysis

Nonfibrillar Aβ, fibrillar Aβ, and the Aβ PET *z* score were compared between *App^NL-G-F^* and APPPS1 mice at different ages by an unpaired Student *t* test. Mean values of each of the 3 readouts from the *App^NL-G-F^* and APPPS1 groups at different ages were subject to a linear regression analysis. The area between the regression plots served as an index of the potential bias in the estimates of Aβ pathology by Aβ PET.

#### Individual-Level Analysis

We applied regression models using the Aβ PET *z* score of all investigated mice in both models as an outcome variable. Fibrillar Aβ and nonfibrillar Aβ estimates deriving from all age- and genotype-matched mouse groups were used as predictors, and heterogeneity of individual mice with respect to PET results and age were used as additional covariates. We defined heterogeneity as the deviation of individual mice in each genotype from their group mean at each time point. The regression coefficients for fibrillar Aβ and nonfibrillar Aβ were extracted to calculate their relative contributions to the Aβ PET signal. Bootstrapping was performed with 1,000 random samples.

#### Validation Analysis

The derived regression coefficients were applied to immunohistochemistry analysis of independent samples of APPPS1/Trem2^+/+^ and APPPS1/Trem2^−/−^ mice. The predicted Aβ PET *z* scores were compared with the actual Aβ PET *z* scores in vivo, and the deviation between the predicted and actual Aβ PET *z* scores was compared with separate consideration of both plaque components and sole consideration of fibrillar Aβ. The bias resulting from consideration of only fibrillar Aβ was calculated as a function of longitudinal changes in the Aβ PET signal in the comparison of APPPS1/Trem2^+/+^ and APPPS1/Trem2^−/−^ mice.

## RESULTS

### Separate Quantification of Fibrillar or Nonfibrillar Aβ Plaque Deposition Fails to Explain the Aβ PET Signal

**FIGURE 1.**
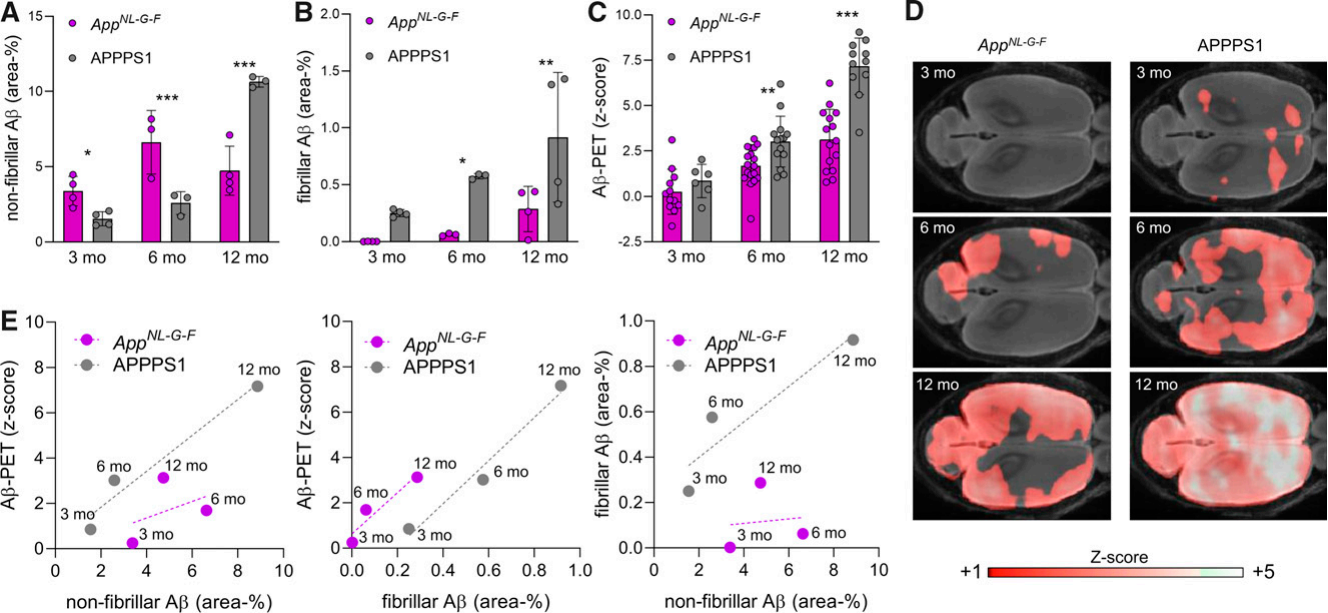
(A–D) Quantitation of nonfibrillar Aβ (A), fibrillar Aβ (B), and Aβ PET signal *z* scores (C) in neocortex of *App^NL-G-F^* and APPPS1 mice at 3, 6, and 12 mo of age, together with axial images of groupwise PET *z* scores projected on MRI standard template (D). (E) Correlation plots of associations between immunochemistry or histochemistry markers and PET at different ages (group level) in comparison of both mouse models. *P < 0.05. **P < 0.01. ***P < 0.001.

**FIGURE 2.**
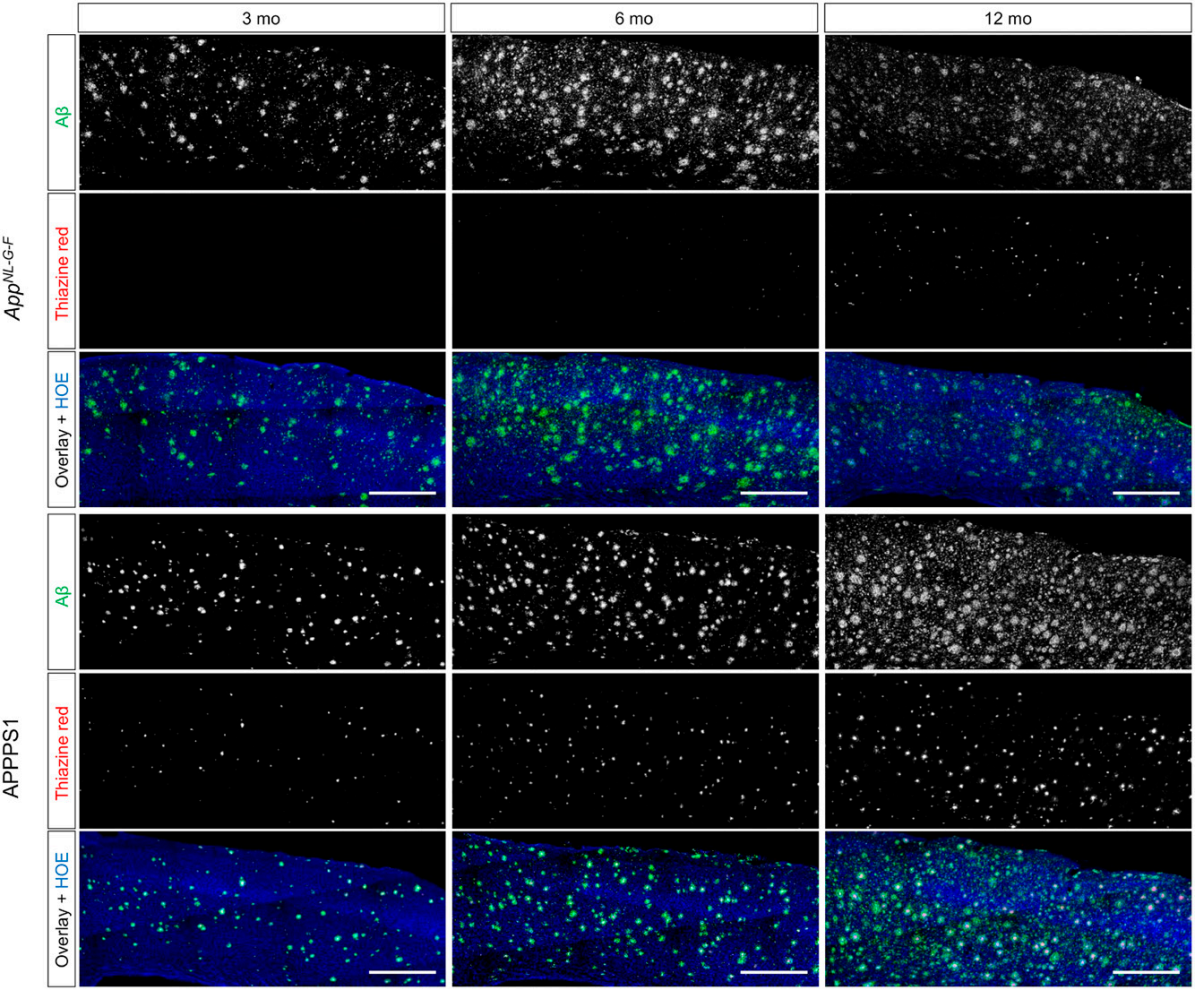
Representative images of immunochemistry and histochemistry. Total Aβ was assessed by 3552 staining, and fibrillar Aβ was assessed by thiazine red. Hoechst (HOE, blue) was used for nuclear visualization (scale bars, 500 μm).

First, we performed a direct standardized comparison of nonfibrillar and fibrillar Aβ estimates by immunohistochemistry and Aβ PET between *App^NL-G-F^* and APPPS1 mouse models at different ages. Nonfibrillar Aβ area coverage of *App^NL-G-F^* mice exceeded that of APPPS1 mice at 3 and 6 mo of age, whereas APPPS1 mice had higher nonfibrillar Aβ area coverage at 12 mo of age (Figs. 1A and 2). Fibrillar Aβ area coverage was significantly higher in APPPS1 mice than in *App^NL-G-F^* mice at all ages studied (Figs. 1B and 2). Immunohistochemically assessed area coverage values did not differ between the immunohistochemistry cohorts and the PET cohorts at 12/13 mo of age (all *P* > 0.05, Supplemental Fig. 1; supplemental materials are available at http://jnm.snmjournals.org). Aβ PET *z* scores of *App^NL-G-F^* and APPPS1 mice are provided and illustrated in Figures 1C and 1D. There were no interindividual SUV ratio differences between mice imaged 3 times, at 3, 6, and 12 mo, and mice imaged only twice, at 6 and 12 mo (all *P* > 0.05, Supplemental Fig. 2). Aβ PET showed significantly higher standardized differences in APPPS1 mice than in *App^NL-G-F^* mice at 6 and 12 mo, whereas there were no significant differences at 3 mo of age (Fig. 1C). Plotting of Aβ PET results as a linear function of nonfibrillar or fibrillar Aβ at different ages indicated a mismatch between the 2 mouse models (Fig. 1E). Plotting of fibrillar Aβ as a linear function of nonfibrillar Aβ coverage underpinned that APPPS1 mice had a higher proportion of fibrillar Aβ than did *App^NL-G-F^* mice (Fig. 1E). The comparison of the linear functions of both mouse models (the area transected by the regression lines) indicated that Aβ PET underestimated the proportion of nonfibrillar Aβ in *App^NL-G-F^* mice (−2.08 *z* score units) but overestimated the proportion of fibrillar Aβ in *App^NL-G-F^* mice (+2.36 *z* score units). Thus, neither fibrillar nor nonfibrillar Aβ alone could explain the combined Aβ PET findings.

### Nonfibrillar Aβ Contributes Significantly to the Aβ PET Signal

Next, we hypothesized that a combined model of nonfibrillar and fibrillar Aβ components could improve the explanation of variance in the Aβ PET signals. To test this hypothesis, we established a multiple-regression model using all available combinations of age- and genotype-matched Aβ PET–immunochemistry or histochemistry estimates with inclusion of all *App^NL-G-F^* and APPPS1 mice.

**FIGURE 3.**
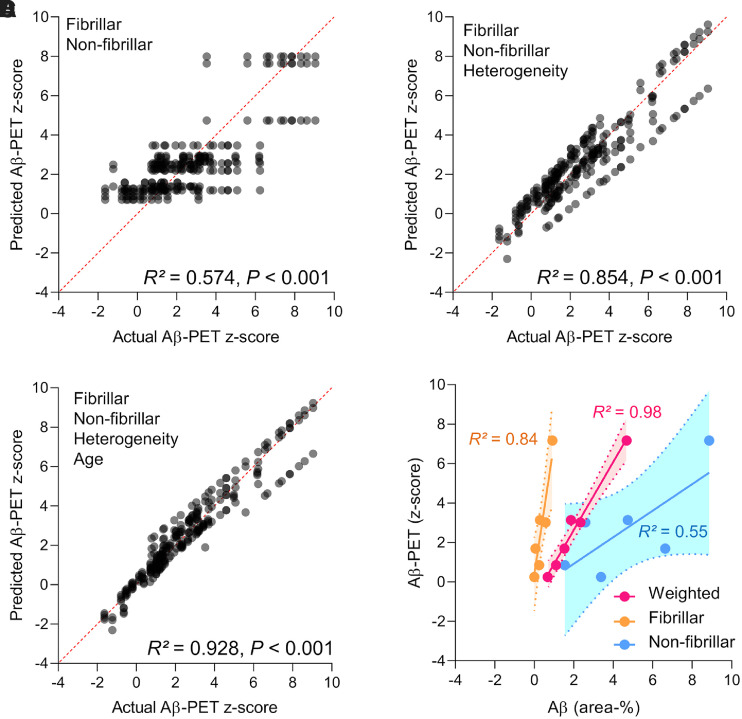
(A–C) Regression plots illustrate correlation between actual and predicted Aβ PET *z* score when using fibrillar Aβ and nonfibrillar Aβ as predictors and using individual heterogeneity and age as covariates. Regressions were calculated with total of 261 permutations between immunochemistry or histochemistry and PET endpoints using all available combinations with matched age and genotype. (D) Application of average regression factors for fibrillar (B = 3.17) and nonfibrillar (B = 0.20) Aβ on combined immunochemistry and histochemistry data for both models (group means per age).

Simplified regression models with either fibrillar or nonfibrillar Aβ as predictors of the Aβ PET *z* score explained 50% and 32% of the variance in Aβ PET, respectively (both *P* < 0.001, Table 1). Combined consideration of fibrillar Aβ and nonfibrillar Aβ as predictors of the Aβ PET *z* score increased the explained variance to 57% (*P* < 0.001, Table 1; Fig. 3A), and fibrillar (β = 0.563, *P* = 1.11e^−27^) and nonfibrillar (β = 0.309, *P* = 9.38e^−11^) Aβ were both strong and independent predictors of the Aβ PET *z* score.

**TABLE 1:** Coefficients of Determination and Regression Coefficients for Prediction of Aβ PET Signal by Fibrillar and Nonfibrillar Aβ Components, with Additional Factoring for Heterogeneity (in Mice with Equal Genotype at Single Time Point) and Age

Model	*R* ^2^	Corrected *R*^2^	B Fibrillar	B Nonfibrillar	*P*
Fibrillar	0.499	0.497	4.414 (3.915–4.926)		<0.001
Nonfibrillar	0.324	0.322		0.477 (0.378–0.569)	<0.001
Fibrillar × nonfibrillar	0.574	0.571	3.521 (2.954–4.041)	0.259 (0.178–0.341)	<0.001
Fibrillar × nonfibrillar × heterogeneity	0.854	0.852	3.521 (2.954–4.041)	0.259 (0.178–0.341)	<0.001
Fibrillar × nonfibrillar × heterogeneity × age	0.928	0.927	2.810 (2.620–3.032)	0.146 (0.103–0.193)	<0.001

*R*^2^ = coefficient of determination; B = regression coefficient.

Numbers in parentheses represent 95% CIs as assessed by bootstrapping with 1,000 random samples.

A model including fibrillar and nonfibrillar Aβ components with the estimate of individual heterogeneity yielded 85% explanation of variance of the Aβ PET signal (Fig. 3B), and further inclusion of age further increased the explanation of variance of the Aβ PET signal (93%, Fig. 3C). Thus, age-related factors influence, importantly, immunochemistry/histochemistry and PET signals (i.e., age-dependent perfusion or partial-volume effects). We considered “fibrillar × nonfibrillar × heterogeneity” and “fibrillar × nonfibrillar × heterogeneity × age” to be the most accurate models, and we calculated the mean regression coefficients from these 2 models to obtain the contributions of fibrillar Aβ and nonfibrillar Aβ to the PET signal. One percent area covered by fibrillar Aβ explained 3.17 PET *z* score units, and 1% area covered by nonfibrillar Aβ explained 0.20 PET *z* score units, thus indicating a 16-fold higher contribution of fibrillar than of nonfibrillar Aβ. The opposite edges of the 95% CIs, as assessed by bootstrapping, indicated a possible range between 11-fold and 26-fold for the relationship between fibrillary and nonfibrillar contributions to the Aβ PET signal. Application of this multiplicative factor to the direct comparison of group-averaged immunochemistry/histochemistry and Aβ PET scores in *App^NL-G-F^* and APPPS1 mice at different ages confirmed the suitability of this model, as indicated by 98% explanation of the variance using weighted factors, compared with only 84% for isolated fibrillar and 55% for nonfibrillar plaque components (Fig. 3D).

### Fibrillar and Nonfibrillar Plaque Components Have an Impact on Mice with Dysfunctional Microglia

**FIGURE 4.**
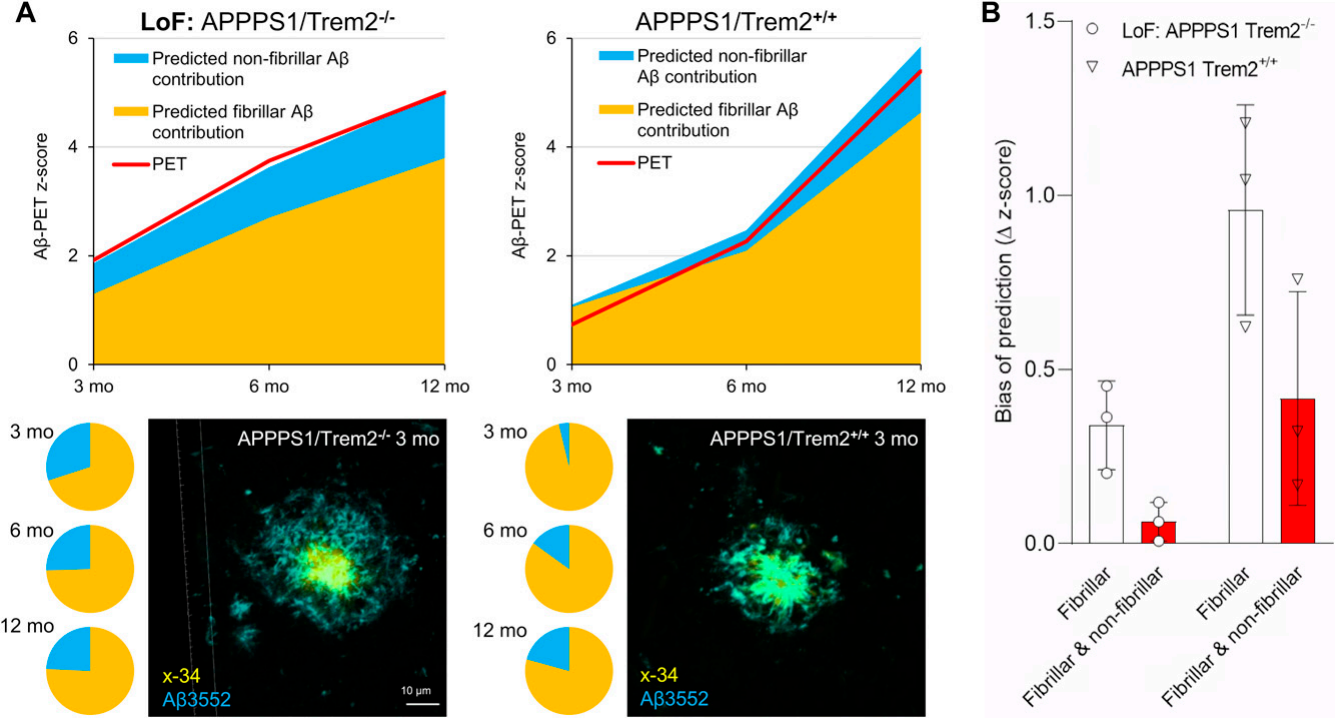
(A) *z* scores of measured Aβ PET signal and predicted fibrillar and nonfibrillar (blue) source components in independent cohort of APPPS1 mice with dysfunctional (Trem2^−/−^) and intact (Trem2^+/+^) microglia. Pie charts show fibrillar and nonfibrillar contributions to measured Aβ PET signals predicted by immunochemistry and histochemistry. Representative double staining of APPPS1/Trem2^−/−^ mouse shows more nonfibrillar Aβ (3552-positive) surrounding core (x-34-positive) than in APPPS1/Trem2^+/+^ mouse, both at 3 mo of age. (B) Bias of predicted *z* scores when only fibrillar or combination of fibrillar and nonfibrillar plaque contributions was considered. Analysis displays deviation of immuno/histochemically predicted Aβ PET values and actual Aβ PET signal. Each dot represents one age-related group of APPPS1/Trem2^−/−^ or APPPS1/Trem2^+/+^ mice (3 mo, 6 mo, 12 mo).

Finally, we validated our results in independent cohorts of APPPS1 mice and made an additional investigation of the impact of Trem2 deficiency on the Aβ PET signal in these mice, given that Trem2 is a known driver of changes in the plaque fibrillarity. Application of the regression factors to immunochemistry/histochemistry data indicated an excellent prediction of the actual Aβ PET signal in independent cohorts of APPPS1/Trem2^−/−^ and APPPS1/Trem2^+/+^ mice (Fig. 4A). APPPS1 mice with Trem2 loss of function showed a higher contribution of nonfibrillar plaque components to the Aβ PET signal (30% at 3 mo, 26% at 6 mo, and 24% at 12 mo) than did APPPS1 mice with intact Trem2 (4% at 3 mo, 15% at 6 mo, and 21% at 12 mo; Fig. 4A). A combined consideration of fibrillar Aβ and nonfibrillar Aβ predicted the actual PET signal more precisely that did sole consideration of fibrillar Aβ (Fig. 4B). Previously calculated increases in Aβ PET signal with age in these mice indicated a considerable bias when considering only the fibrillar Aβ component (Supplemental Fig. 3). In summary, microglial dysfunction altered the relative proportions of fibrillar and nonfibrillar Aβ, thus directly influencing the Aβ PET signal as a function of mouse age.

## DISCUSSION

We provide the first—to our knowledge—in vivo analysis to elucidate the contributions of fibrillar and nonfibrillar plaque components to the Aβ PET signal. Our data clearly show that nonfibrillar plaque fractions have a significant impact on the net ^18^F-florbetaben binding to Aβ plaques of Aβ mouse models in vivo. Although the resulting Aβ PET tracer signal is inherently 16-fold higher when comparing equal amounts of fibrillar and nonfibrillar Aβ, the larger proportions of nonfibrillar plaque components counterbalance the net contribution. We validated our regression model in an independent cohort of APPPS1 mice and extended the potential translational impact of our findings by showing that microglial dysfunction can influence the longitudinal Aβ PET signal via changing the relative proportions of fibrillar-to-nonfibrillar plaque components.

In various analyses of single amyloidosis mouse models, there was a strong agreement between Aβ PET and different immunochemistry or histochemistry markers for Aβ (*9*,*20*). It is widely acknowledged that the Aβ PET signal with ^18^F-florbetaben derives primarily from aggregated fibrillar Aβ, since this and other clinically approved Aβ PET tracers were derived from the chemical scaffold of thioflavin-T, which colors only fibrillar Aβ (*21*). However, a recent small-animal Aβ PET investigation from our lab (*18*) indicated that there could be discrepancies between immunochemistry/histochemistry and in vivo Aβ PET signals, if one attributes the entire PET signal to fibrillar Aβ. In fact, *App^NL-G-F^* mice exhibited an only moderate Aβ PET signal, although their plaques were composed mainly of nonfibrillar Aβ (*14*). Therefore, we applied in this study a standardized ^18^F-florbetaben PET examination comparing *App^NL-G-F^* and APPPS1 mice in conjunction with combined histochemical and immunohistochemical examination to elucidate the separate contributions of fibrillar and nonfibrillar Aβ sources to the in vivo Aβ PET signal. We performed PET acquisitions in both Aβ models and wild-type mice with identical housing conditions and using the same tomograph and image reconstruction parameters, thus minimizing the potential methodologic bias. Nonetheless, we acknowledge that scanning of mice on different days of the week, along with social hierarchy factors and technical factors due to different cage positions, might still impact the detection of plaque pathology by Aβ PET. We had to choose between conducting a longitudinal PET examination with immunochemistry/histochemistry in a seperate cohort and conducting a cross-sectional study in which PET examination directly preceded immunohistochemistry/histochemistry in the same mice. Since animal-batch effects may introduce a bias into cross-sectional PET quantifications between different ages of a given mouse model, we elected to conduct longitudinal PET imaging together with cross-sectional immunochemistry and histochemistry to exclude batch effects, at least for PET. To account further for the heterogeneity (*22*) and asymmetry (*13*) of amyloidosis in individual mice of a lineage, we used a bilateral target in a regression model including each available combination of PET–immunochemistry/histochemistry results for each model at each of 3 ages, controlled for the individual heterogeneity. Regression coefficients for the proportions of fibrillar and nonfibrillar Aβ in different models were robust and revealed that fibrillar Aβ makes an intrinsically 16-fold higher contribution to the Aβ PET signal than nonfibrillar Aβ in the studied Aβ mouse models. Our preclinical in vivo results concur with the postmortem validation of human ^18^F-flutemetamol PET data, where ligand binding to diffuse plaques was the most likely explanation for positive in vivo signals in patients who later proved to have only sparse neuritic plaques at autopsy (*11*,*23*). The same research group recently validated the contributions of diffuse and neuritic plaques to the ^18^F-flutemetamol and ^11^C-Pittsburgh compound B autoradiography signals in an in vitro study (*12*). However, our study was the first translation of such findings into the in vivo setting and enables the arithmetic conversion of Aβ PET signals into fibrillar and nonfibrillar Aβ components. We note that the structure of the stilbene ^18^F-florbetaben is different from the structures of the benzothiazoles ^18^F-flutemetamol and ^11^C-Pittsburgh compound B and that this difference could result in different proportions of fibrillar and nonfibrillar binding capacities in vivo. As usual, the limited resolution of small-animal PET systems in relation to the mouse brain size and resulting partial-volume effects present a limitation for the transfer of the present findings into the human context, and we want to emphasize that detailed regression factors cannot be transferred directly. Yet, the demonstration of an inherently 16-fold higher contribution of fibrillar plaque to the PET signal concurs with a biophysical chemistry study investigating binding mechanisms of Aβ ligands by molecular docking, molecular dynamics, and generalized Born-based free-energy calculations (*24*). Here, core sites of Aβ fibrils, which are more abundant in fibrillar components of the plaque, dominated over surface sites in producing the Aβ PET signal (*24*).

Our findings could be of translational relevance since Aβ-immunotherapy and other treatment strategies against AD may change the proportions of fibrillar and nonfibrillar plaque components and thus bias the Aβ PET outcome. Furthermore, alterations of microglial genes are associated with changes in plaque morphology, which consequently influence the Aβ PET signal (*5*). We can correlate the more diffuse amyloid plaque morphology in *App^NL-G-F^* mice with differences in plaque morphology observed in AD mice deficient in *TREM2* or *APOE* (*5*,*25*). Although loss-of-function mutations of Trem2 are rare in humans, microglia genes seem in general (*26*) to have a high impact on AD pathology, and modulation of microglial function is being intensively studied as a therapeutic strategy for AD (*27*). Another limitation for the direct translation of our findings toward human AD consists in different binding site densities of Aβ plaques in Aβ mouse models when compared with sporadic AD (*28*). Thus, comparisons of Aβ PET signal intensity between rodents and humans need to be considered with caution. However, the regression model generated in this study should increase awareness of the impact of nonfibrillar Aβ on the Aβ PET signal in both species. Thus, a potential shift in the plaque proportions needs to be considered when designing future Aβ PET monitored studies that target microglia. Assessment of fibrillar and nonfibrillar plaque components and the respective Aβ PET tracer–binding properties in autopsy cases after or during disease-modifying treatment studies of AD could serve to test the impact of our findings on the human situation.

## CONCLUSION

The Aβ PET signal with ^18^F-florbetaben in vivo arises from a combination of fibrillar and nonfibrillar plaque components. Fibrillar Aβ has inherently higher tracer binding, but the greater proportion of nonfibrillar Aβ relative to fibrillar Aβ in most plaques means that the nonfibrillar signal source is a relevant component of the total signal. Since experimental AD therapy regimens can shift the proportion of fibrillar versus nonfibrillar Aβ, any longitudinal changes in Aβ PET signal as a readout of therapy monitoring must be interpreted with caution; a detailed understanding of the biochemical basis of Aβ PET signal is critical for the correct use of PET for monitoring novel AD therapies.

## DISCLOSURE

This work was supported by an Alzheimer’s Association grant through the AD Strategic Fund (ADSF-21-831226-C) and by the German Research Foundation within the framework of the Munich Cluster for Systems Neurology (EXC 2145 SyNergy/ID 390857198). Sabina Tahirovic was supported by the Alzheimer-Forschung-Initiative e.V (grant 18014). Christian Haass collaborates with Denali Therapeutics, participated in one advisory board meeting of Biogen, and received a speaker honorarium from Novartis and Roche. Christian Haass is chief advisor of ISAR Bioscience. Peter Bartenstein, Axel Rominger, and Matthias Brendel received speaking honoraria from Life Molecular Imaging and GE Healthcare. Matthias Brendel is an advisor of Life Molecular Imaging. No other potential conflict of interest relevant to this article was reported.

## Notes

KEY POINTS **QUESTION:** Does nonfibrillar Aβ contribute to the in vivo Aβ PET signal? **PERTINENT FINDINGS:** Fibrillar Aβ has 16-fold higher tracer binding than does nonfibrillar Aβ, but the greater proportion of nonfibrillar Aβ relative to fibrillar Aβ in most plaques means that the nonfibrillar signal source is a relevant component of the total Aβ PET signal. **IMPLICATIONS FOR PATIENT CARE:** Shifts of the proportion of fibrillar versus nonfibrillar Aβ need to be considered when interpreting the longitudinal Aβ PET signal for monitoring of therapeutic effects.

## References

[1] Koscik RL Betthauser TJ Jonaitis EM.Amyloid duration is associated with preclinical cognitive decline and tau PET. Alzheimers Dement (Amst). 2020;12:e12007. 32211502 10.1002/dad2.12007 PMC7085284

[2] Jack CR Jr Bennett DA Blennow K.NIA-AA research framework: toward a biological definition of Alzheimer’s disease. Alzheimers Dement. 2018;14:535–562. 29653606 10.1016/j.jalz.2018.02.018 PMC5958625

[3] Wessels AM Tariot PN Zimmer JA.Efficacy and safety of lanabecestat for treatment of early and mild Alzheimer disease: the AMARANTH and DAYBREAK-ALZ randomized clinical trials. JAMA Neurol. 2020;77:199–209. 31764959 10.1001/jamaneurol.2019.3988 PMC6902191

[4] Sevigny J Chiao P Bussière T.The antibody aducanumab reduces Aβ plaques in Alzheimer’s disease. Nature. 2016;537:50–56. 27582220 10.1038/nature19323

[5] Parhizkar S Arzberger T Brendel M.Loss of TREM2 function increases amyloid seeding but reduces plaque-associated ApoE. Nat Neurosci. 2019;22:191–204. 30617257 10.1038/s41593-018-0296-9 PMC6417433

[6] Overhoff F Brendel M Jaworska A.Automated spatial brain normalization and hindbrain white matter reference tissue give improved [^18^F]-florbetaben PET quantitation in Alzheimer’s model mice. Front Neurosci. 2016;10:45. 26973442 10.3389/fnins.2016.00045 PMC4770021

[7] Sabri O Sabbagh MN Seibyl J.Florbetaben PET imaging to detect amyloid beta plaques in Alzheimer’s disease: phase 3 study. Alzheimers Dement. 2015;11:964–974. 25824567 10.1016/j.jalz.2015.02.004

[8] Curtis C Gamez JE Singh U.Phase 3 trial of flutemetamol labeled with radioactive fluorine 18 imaging and neuritic plaque density. JAMA Neurol. 2015;72:287–294. 25622185 10.1001/jamaneurol.2014.4144

[9] Rominger A Brendel M Burgold S.Longitudinal assessment of cerebral b-amyloid deposition in mice overexpressing Swedish mutant b-amyloid precursor protein using ^18^F-florbetaben PET. J Nucl Med. 2013;54:1127–1134. 23729696 10.2967/jnumed.112.114660

[10] Catafau AM Bullich S Seibyl JP.Cerebellar amyloid-beta plaques: how frequent are they, and do they influence ^18^F-florbetaben SUV ratios? J Nucl Med. 2016;57:1740–1745. 27363836 10.2967/jnumed.115.171652

[11] Ikonomovic MD Buckley CJ Heurling K.Post-mortem histopathology underlying beta-amyloid PET imaging following flutemetamol F 18 injection. Acta Neuropathol Commun. 2016;4:130. 27955679 10.1186/s40478-016-0399-z PMC5154022

[12] Ikonomovic MD Buckley CJ Abrahamson EE.Post-mortem analyses of PiB and flutemetamol in diffuse and cored amyloid-beta plaques in Alzheimer’s disease. Acta Neuropathol (Berl). 2020;140:463–476. 32772265 10.1007/s00401-020-02175-1 PMC7498488

[13] Sacher C Blume T Beyer L.Asymmetry of fibrillar plaque burden in amyloid mouse models. J Nucl Med. 2020;61:1825–1831. 32414948 10.2967/jnumed.120.242750

[14] Sebastian Monasor L Muller SA Colombo AV.Fibrillar Abeta triggers microglial proteome alterations and dysfunction in Alzheimer mouse models. eLife. 2020;9:e54083. 10.7554/eLife.54083 PMC7279888 32510331

[15] Radde R Bolmont T Kaeser SA.Abeta42-driven cerebral amyloidosis in transgenic mice reveals early and robust pathology. EMBO Rep. 2006;7:940–946. 16906128 10.1038/sj.embor.7400784 PMC1559665

[16] Masuda A Kobayashi Y Kogo N Saito T Saido TC Itohara S. Cognitive deficits in single App knock-in mouse models. Neurobiol Learn Mem. 2016;135:73–82. 27377630 10.1016/j.nlm.2016.07.001

[17] Saito T Matsuba Y Mihira N.Single App knock-in mouse models of Alzheimer’s disease. Nat Neurosci. 2014;17:661–663. 24728269 10.1038/nn.3697

[18] Sacher C Blume T Beyer L.Longitudinal PET monitoring of amyloidosis and microglial activation in a second-generation amyloid-beta mouse model. J Nucl Med. 2019;60:1787–1793. 31302633 10.2967/jnumed.119.227322 PMC6894380

[19] Yamasaki A Eimer S Okochi M.The GxGD motif of presenilin contributes to catalytic function and substrate identification of gamma-secretase. J Neurosci. 2006;26:3821–3828. 16597736 10.1523/JNEUROSCI.5354-05.2006 PMC6674133

[20] Poisnel G Dhilly M Moustie O.PET imaging with [^18^F]AV-45 in an APP/PS1-21 murine model of amyloid plaque deposition. Neurobiol Aging. 2012;33:2561–2571. 22277262 10.1016/j.neurobiolaging.2011.12.024

[21] Mathis CA Mason NS Lopresti BJ Klunk WE. Development of positron emission tomography beta-amyloid plaque imaging agents. Semin Nucl Med. 2012;42:423–432. 23026364 10.1053/j.semnuclmed.2012.07.001 PMC3520098

[22] Brendel M Jaworska A Herms J.Amyloid-PET predicts inhibition of de novo plaque formation upon chronic gamma-secretase modulator treatment. Mol Psychiatry. 2015;20:1179–1187. 26055427 10.1038/mp.2015.74 PMC4759098

[23] Ikonomovic MD Fantoni ER Farrar G Salloway S. Infrequent false positive [^18^F]flutemetamol PET signal is resolved by combined histological assessment of neuritic and diffuse plaques. Alzheimers Res Ther. 2018;10:60. 29935545 10.1186/s13195-018-0387-6 PMC6015459

[24] Murugan NA Halldin C Nordberg A Langstrom B Agren H. The culprit is in the cave: the core sites explain the binding profiles of amyloid-specific tracers. J Phys Chem Lett. 2016;7:3313–3321. 27498616 10.1021/acs.jpclett.6b01586

[25] Ulrich JD Ulland TK Mahan TE.ApoE facilitates the microglial response to amyloid plaque pathology. J Exp Med. 2018;215:1047–1058. 29483128 10.1084/jem.20171265 PMC5881464

[26] Sierksma A Lu A Mancuso R.Novel Alzheimer risk genes determine the microglia response to amyloid-beta but not to TAU pathology. EMBO Mol Med. 2020;12:e10606. 31951107 10.15252/emmm.201910606 PMC7059012

[27] Lewcock JW Schlepckow K Di Paolo G Tahirovic S Monroe KM Haass C. Emerging microglia biology defines novel therapeutic approaches for Alzheimer’s disease. Neuron. 2020;108:801–821. 33096024 10.1016/j.neuron.2020.09.029

[28] Klunk WE Lopresti BJ Ikonomovic MD.Binding of the positron emission tomography tracer Pittsburgh compound-B reflects the amount of amyloid-beta in Alzheimer’s disease brain but not in transgenic mouse brain. J Neurosci. 2005;25:10598–10606. 16291932 10.1523/JNEUROSCI.2990-05.2005 PMC6725842

